# The neuroprotective action of 3,3′-diindolylmethane against ischemia involves an inhibition of apoptosis and autophagy that depends on HDAC and AhR/CYP1A1 but not ERα/CYP19A1 signaling

**DOI:** 10.1007/s10495-019-01522-2

**Published:** 2019-02-18

**Authors:** J. Rzemieniec, A. Wnuk, W. Lasoń, W. Bilecki, M. Kajta

**Affiliations:** 1 Department of Experimental Neuroendocrinology, Laboratory of Molecular Neuroendocrinology, Institute of Pharmacology, Polish Academy of Sciences, Smetna Street 12, 31-343 Krakow, Poland; 2 Department of Experimental Neuroendocrinology, Institute of Pharmacology, Polish Academy of Sciences, Smetna Street 12, 31-343 Krakow, Poland; 3 Department of Pharmacology, Laboratory of Pharmacology and Brain Biostructure, Institute of Pharmacology, Polish Academy of Sciences, Smetna Street 12, 31-343 Krakow, Poland

**Keywords:** 3,3′-Diindolylmethane, Ischemia, Neuroprotection, Apoptosis, Autophagy, AhR

## Abstract

There are no studies examining the effects of 3,3′-diindolylmethane (DIM) in neuronal cells subjected to ischemia. Little is also known about the roles of apoptosis and autophagy as well as AhR and ERα signaling and HDACs in DIM action. We demonstrated for the first time the strong neuroprotective capacity of DIM in mouse primary hippocampal cell cultures exposed to ischemia at early and later stages of neuronal development. The protective effects of DIM were mediated via inhibition of ischemia-induced apoptosis and autophagy that was accompanied by a decrease in AhR/CYP1A1 signaling and an increase in HDAC activity. DIM decreased the levels of pro-apoptotic factors, i.e., Fas, Caspase-3, and p38 mitogen-activated protein kinase (MAPK). DIM also reduced the protein levels of autophagy-related Beclin-1 (BECN1) and microtubule-associated proteins 1A/1B light chain (LC3), partially reversed the ischemia-induced decrease in Nucleoporin 62 (NUP62) and inhibited autophagosome formation. In addition, DIM completely reversed the ischemia-induced decrease in histone deacetylase (HDAC) activity in hippocampal neurons. Although DIM inhibited AhR/CYP1A1 signaling, it did not influence the protein expression levels of ERα and ERα-regulated CYP19A1 which are known to be controlled by AhR. This study demonstrated for the first time, that the neuroprotective action of 3,3′-diindolylmethane against ischemia involves an inhibition of apoptosis and autophagy and depends on AhR/CYP1A1 signaling and HDAC activity, thus creating the possibility of developing new therapeutic strategies that target neuronal degeneration at specific molecular levels.

## Introduction

According to the latest statistics, stroke is the 5th leading cause of death in people aged 15 to 59 years and the 2nd leading cause of death in people over the age of 60 years [1]. Despite extensive experimental and clinical efforts, thrombolysis with recombinant tissue-type plasminogen activator (rt-PA) remains the only FDA-approved clinical treatment for ischemic stroke [2]. In newborns, resuscitation and mild hypothermia may diminish neurological deficits following asphyxia [3]. Unfortunately, both rt-PA and hypothermia have narrow therapeutic window, low levels of specificity and efficacy, and long list of contraindications. Moreover, many neuroprotective agents that showed positive results in experimental models of stroke failed in clinical trials [4, 5]. For that reason, there is a strong need to find a novel effective compound that may protect the brain undergoing ischemia at different stages of development. One of these candidates could be the plant-derived − 3,3′-diindolylmethane (DIM) which is currently in clinical trials and it does not show serious side effects in healthy volunteers [6]. DIM is a representative of selective aryl hydrocarbon receptor modulators (SAhRMs). Recently, it has been shown that expression of aryl hydrocarbon receptor (AhR) and its heterodimerization partner ARNT was substantially increased in neuronal cells and the brain after experimental stroke in vitro and in vivo [7, 8]. We have previously demonstrated that 3,3′-diindolylmethane protects hippocampal cells against hypoxia via inhibition of AhR/ARNT-dependent apoptosis [8]. In addition, we demonstrated that ERα, which is known to interact with AhR, plays a crucial role in protecting neurons against hypoxia [9–12]. However, there are no data showing neuroprotective effects of DIM in ischemia which model not only oxygen but also nutritional deprivations that accompany stroke-induced apoptosis.

DIM is found in cruciferous vegetables including cauliflower, broccoli, brussels sprouts, cabbage and kale. The anti-inflammatory and neuroprotective potential of 3,3′-diindolylmethane and its analogs have been demonstrated in models of Parkinson’s disease and brain inflammation [13–17]. Moreover, there are some data showing that DIM exhibits properties of histone deacetylase (HDAC) inhibitor in prostate and colon cancer cell lines [18, 19]. It is well known that HDAC inhibitors exert neuroprotective properties in experimental models of stroke and reduce stroke risk after previous ischemic stroke in humans [20–25]. Despite these data, the role of HDAC in stroke is still controversial; there is evidence of HDAC stimulation in response to ischemia but there are also data showing inhibition of HDAC in response to ischemia [26–30]. However, the involvement of HDAC in neuroprotective action of DIM in experimental model of stroke remains unknown.

It is generally accepted that neurons subjected to ischemia die of necrosis and apoptosis [31–34]. There are two main pathways of apoptosis - the extrinsic and intrinsic pathways. The extrinsic mechanism of apoptosis involves the activation of cell death receptors such as FasR, establishment of death-inducing signaling complex (DISC), and activation of initiator caspase-8 and effector caspase-3 [35, 36]. The intrinsic pathway includes mitochondrial membrane permeabilization controlled by proteins from the BCL2 family, cytochrome C release, the formation of apoptosomes, and the activation of caspase-9 and caspase-3. It has been demonstrated that neuronal apoptosis might also be mediated by persistent activation of c-Jun NH2-terminal kinase (JNK) or p38 signaling pathways [37]. Other studies have shown that inhibition of ischemia-induced apoptosis and necrosis leads to neuroprotection [38–41]. However, there are no data on the role of DIM in inhibition of ischemia-induced extrinsic and intrinsic apoptotic pathways.

Pathophysiology of stroke-induced neuronal death involves not only necrosis and apoptosis but also autophagy [42–44]. Autophagy is a process consisting of several steps including induction, formation of the phagophores and autophagosomes, fusion with lysosome, and degradation [45]. Many autophagy-related proteins, such as ULK1/2, ATG13, VPS34, Beclin-1, ATG7, LC3 and NUP62, are involved in the propagation of these stages [46]. Nevertheless, the exact role of autophagy during ischemia is not clear. There are many in vitro and in vivo studies showing detrimental effects of autophagy in an experimental stroke [47], but the inhibitory action of DIM on detrimental effects of autophagy during ischemia has not been determined.

This study aimed to assess the neuroprotective capacity of 3,3′-diindolylmethane in an in vitro model of ischemia i.e., oxygen and glucose deprivation (OGD). Particular attention was paid to the influence of DIM on apoptosis, autophagy and on epigenetic enzyme HDAC as well as AhR and ERα signaling pathways in mouse neurons subjected to OGD.

## Materials and methods

### Materials

B27 and Neurobasal media with or without glucose were obtained from Gibco (Grand Island, NY, USA). L-glutamine, fetal bovine serum (FBS), N-acetyl-Asp-Glu-Val-Asp *p*-nitro-anilide (Ac-DEVD-*p*NA), dimethyl sulfoxide (DMSO), HEPES, CHAPS, poly-ornithine and DL-dithiothreitol were obtained from Sigma–Aldrich (St. Louis, MO, USA). The culture dishes were obtained from TPP Techno Plastic Products AG (Trasadingen, Switzerland). 3,3′-Diindolylmethane was purchased from Sigma–Aldrich (St. Louis, MO, USA). A gas cylinder filled with 95% nitrogen and balanced carbon dioxide was rented from Linde Gas (Poland). A BM Chemiluminescence Western Blotting Substrate (POD) and Cytotoxicity Detection Kit (LDH) were purchased from Roche Diagnostics GmbH (Mannheim, Germany). 2x Laemmli Sample Buffer, Bradford reagent, 10% Mini-PROTEAN TGX Precast Gels and 7.5% Mini-PROTEAN TGX Precast Gels were obtained from Bio-Rad Laboratories (Hercules, CA, USA). RIPA buffer, Protease Inhibitor Cocktail for Mammalian Tissues, a Histone Deacetylase Assay Kit, an Autophagy Assay Kit, p38/MAPK, caspase-9, caspase-8 and JNK inhibitors (SB203580, Z-Leu-Glu(OMe)-His-Asp(OMe) fluoromethylketone trifluoroacetate salt hydrate, Z-Leu-Glu(OMe)-Thr-Asp(OMe) fluoromethylketone, SP600125) were bought from Sigma Aldrich (St. Louis, MO, USA). Immobilon-P membranes were obtained from Merck Millipore (Burlington, MA, USA). Mouse monoclonal anti-β-Actin antibody (sc-47778), mouse monoclonal anti-MAP LC3 α/β antibody (sc-398822), rabbit polyclonal anti-BECN1 antibody (sc-11427), rabbit polyclonal anti-AhR antibody (sc-5579), goat polyclonal CYP1A1 antibody (sc-9828), rabbit polyclonal ERα antibody (sc-7207), mouse monoclonal anti-nucleoporin antibody p62 (sc-48373), mouse monoclonal anti-p-p38 antibody (Tyr 182) (sc-166182), rabbit polyclonal anti-Fas antibody (sc-1023), rabbit polyclonal anti-Bax antibody (sc-493), mouse monoclonal anti-Bcl2 antibody (sc-7382) were purchased from Santa Cruz Biotechnology, Inc. (Santa Cruz, CA, USA). Cy3-conjugated anti-mouse IgG, Cy3-conjugated anti-rabbit IgG, Cy5-conjugated anti-mouse IgG, Cy3-conjugated anti-goat IgG were bought from Jackson Immunoresearch Laboratories Inc. (West Grove, PA, USA). Anti-rabbit cleaved caspase-3 antibody (9661) and anti-rabbit Atg7 antibody (2631) were obtained from Cell Signaling Technology (Danvers, Massachusetts, USA). Mouse anti-human CYP19A1 antibody (MCA 2077S) was purchased from Bio-Rad Laboratories (Hercules, CA, USA). NeuroFluor™ NeuO was obtained from Stemcell Technologies (Vancouver, Canada) and Hoechst 33342 was purchased from Molecular Probes (Eugene, OR, USA). ELISA kits for BECN1 and NUP62 were purchased from Shanghai Sunred Biological Technology Co. (Sunred, China).

### Primary hippocampal and neocortical cell cultures

The primary hippocampal and neocortical cell cultures were established from Swiss CD1 mouse embryos (Charles River, Sulzfeld, Germany) at 15–17 days of gestation and were cultured in phenol red-free Neurobasal medium with 5% FBS as previously described [12, 48]. The neuronal cells were seeded onto multiwell plates pre-coated with poly-ornithine at a density of 2.5 × 10^5^ cells per cm^2^. After 2 days, the medium was changed with FBS-free Neurobasal supplemented with B27 and L-glutamine. The cells were maintained at 37 ^ο^C in incubator containing 5% CO_2_ for 2, 7, and 12 days in vitro (DIV) before experiments. The fraction of astrocytes was approximately 10%, as determined previously [49]. In this study, all efforts were made to minimize the number and suffering of mice. All procedures were performed in accordance with the Guidelines for the Care and Use of Laboratory Animals from the National Institutes of Health, ANNEX IV of DIRECTIVE 2010/63/EU and were accepted by the Bioethics Commission in compliance with Polish Law (21 August 1997).

### Treatment and experimental model of ischemia

The mouse hippocampal cell cultures were treated with DIM (0.01, 0.1, 1, 10 µM) just prior to oxygen and glucose deprivation on DIV 2, 7 and 12. The ischemic conditions were evoked by changing the medium to Neurobasal medium without glucose, relocating the plates into a humidified hypoxic modular incubator chamber (Billups-Rothenberg, Del Mar, CA) and then flushing the chamber with 95% N_2_/5% CO_2_ for 6 min to obtain an O_2_ gas pressure around zero. After 6 h of OGD, the medium was changed, and the cells were transferred in the incubator (37 °C, 19% O_2_, 5% CO_2_) for the next 18 h (reoxygenation). For assessment of apoptotic signaling, we used a cell-permeable p38 MAPK inhibitor (SB203580; 1 µM), JNK inhibitor (SP600125; 1 µM), and caspase-8 (Z-LETD-FMK; 40 µM) and caspase-9 (Z-LEHD-FMK; 40 µM) inhibitors simultaneously with DIM. In our study, the DIM and inhibitors of caspases and kinases were used at concentrations that did not change the control levels of caspase-3 activity and LDH release under normoxic conditions to avoid nonspecific effects. According to our data, concentrations of DIM greater than 10 µM were toxic to the cell cultures maintained under normoxic conditions [8]. Therefore, we only used concentrations of 10 µM and less than 10 µM to study the neuroprotective effects of DIM on ischemia-induced cell damage. DIM was originally dissolved in DMSO and then further diluted in culture medium, resulting in DMSO concentrations of less than 0.1%.

### Identification of apoptotic cells

Nuclear condensation can be used to distinguish apoptotic cells from healthy cells or necrotic cells. Cells with condensed nuclei were detected via Hoechst 33342 staining after 6 h of ischemia followed by 18 h of reoxygenation. Hippocampal neurons were cultured on glass coverslips in 24-well plates. After 7 DIV, the cells were subjected to DIM (10 µM) and OGD (6 h). After 18 h of reoxygenation the neurons were washed with phosphate-buffered saline (PBS) and stained with Hoechst 33342 (0.6 mg/ml) at room temperature (RT) for 5 min, as previously described [50]. The cells with bright blue fragmented nuclei, which were indicative of condensed chromatin, were identified as apoptotic cells. The images were prepared using an inverted fluorescence microscope (AxioObserver, Carl Zeiss) with an excitation wavelength of 490 nm (UV fluorescence) and by the use of Axiovision 3.1 software (Carl Zeiss, Germany).

### Staining with NeuroFluor™ NeuO

NeuO is a membrane-permeable fluorescent probe that selectively stains live neurons. The hippocampal cells were cultured on glass coverslips. The neurons were subjected to DIM and ischemia and then incubated with 0.125 µM NeuO suspended in Neurobasal for 2 h. Then, the labeling medium was replaced with fresh medium, and the cells were incubated for another 2 h. Fluorescence images were recorded and developed using an inverted fluorescence microscope (AxioObserver, Carl Zeiss) with an excitation wavelength of 350 nm (UV fluorescence) and Axiovision 3.1 software (Carl Zeiss, Germany).

### Determination of Caspase-3 Activity

Caspase-3 activity was measured in neurons that had been simultaneously treated with DIM (0.01-10 µM) and subjected to 6 h ischemia followed by 18 h of reoxygenation using the methods reported by [51]. The cells were disintegrated with the use of lysis buffer and then incubated at 37 °C with the colorimetric substrate Ac-DEVD-*p*NA, which is cleaved by caspase-3. The levels of *p*-nitroanilide were measured after 60 min using an Infinite M200PRO microplate reader (Tecan, Mannedorf, Switzerland). The data were analyzed using the i-control software, standardized to the DMSO-treated cells, and expressed as the percentage of control ± SEM of three independent experiments. The blank sample was subtracted from each value.

### Measurement of LDH activity

LDH release from the cells into the culture media in response to ischemia and DIM (0.01–10 µM) was measured to quantify cell death as previously described [52]. After experiment, the supernatants were collected from each well, placed to a new dish, and incubated with the reagent mixture at RT for 30 min, according to the manufacturer’s instructions (Cytotoxicity Detection Kit). The red color created during the chemical reaction was measured at a wavelength of 490 nm and was proportional to the LDH activity released from damaged cells. The data were normalized to the DMSO-treated cells and expressed as a percentage of the control from three independent experiments.

### Western blot analyses

The hippocampal cells were cultured in 6-well plates at a density of 1.75 × 10^6^ cells. After the experiment, the neurons were lysed in cold RIPA lysis buffer containing protease inhibitor cocktail. The lysates were sonicated and centrifuged at 15,000×*g* at 4 °C for 20 min. The protein concentrations in the supernatants were determined using Bradford reagent (BioRad Protein Assay) with bovine serum albumin (BSA) as the standard. Samples containing 30 µg of total protein were reconstituted in the appropriate amount of Laemmli sample buffer, denatured (95 °C, 5 min), and separated on 7.5 and 10% SDS–polyacrylamide gels using a Bio-Rad Mini-Protean 3 system as previously described [12]. After electrophoresis, the proteins were subjected to 60 min transfer onto PVDF membranes (Merck Millipore) using a Bio-Rad Mini Trans-Blot apparatus. Afterwards, the nonspecific binding sites were blocked with 5% nonfat dry milk and 0.2% Tween-20 in 0.02 M TBS (Tris-buffered saline) for 1.5 h with shaking. Then, the membranes were incubated overnight (at 4 °C) with one of the following primary antibodies (Santa Cruz Biotechnology): mouse monoclonal anti-β-Actin antibody (diluted 1:3000), mouse monoclonal anti-MAP LC3 α/β (diluted 1:150), rabbit polyclonal anti-BECN-1 antibody (diluted 1:100), rabbit polyclonal anti-AhR antibody (diluted 1:300), rabbit polyclonal anti-ERα antibody (diluted 1:300), mouse monoclonal anti-CYP19A1 (1:200), goat polyclonal anti-CYP1A1 (1:200), mouse monoclonal anti-nucleoporin p62 antibody (diluted 1:1000), mouse monoclonal anti-p-p38 antibody (diluted 1:50), rabbit polyclonal anti-Fas antibody (diluted 1:100), rabbit polyclonal anti-BAX antibody (diluted 1:100), rabbit polyclonal anti-cleaved Caspase-3 antibody (diluted 1:1000), and rabbit monoclonal anti-ATG7 antibody (diluted 1:1000) diluted in TBS/Tween. Subsequently, the membranes were washed 5 times with 4% nonfat milk with TBS and 0.2% Tween 20 and incubated for 1 h with horseradish peroxidase-conjugated secondary antibodies (goat anti-rabbit IgG or goat anti-mouse IgG) diluted at 1:1000 or/and 1:3000, in 0.25% non-fat milk with TBS/Tween. The images were developed using BM Chemiluminescence Blotting Substrate (Roche Diagnostics GmBH) and visualized using a Luminescent Image Analyzer Fuji-Las 4000 (Fuji, Japan). The immunoreactive bands were quantified using an image analyzer (ScienceLab, MultiGauge V3.0).

### ELISAs for NUP62 and BECN1

The levels of NUP62 and BECN1 were determined via ELISA (Shanghai Sunred Biological Technology Co., Ltd. Shanghai, China) directly after ischemia and after 18 h of reoxygenation. Detection of these proteins was achieved using a commercially available quantitative sandwich enzyme immunoassay kits. Specific monoclonal antibodies to NUP62 and BECN1 were precoated on a 96-well plate. Standards and cell extracts were added to the wells containing biotin-conjugated polyclonal antibodies specific to NUP62 and BECN1. Thus, NUP62 and BECN1 were caught by the antibodies. Following a wash to remove any unbound substances, horseradish peroxidase-conjugated avidin was added to interact with the biotin bound to NUP62 and BECN1. Following another wash, a substrate solution was added to the wells. The enzyme reaction produced a blue product. The absorbance was measured at 450 nm and was proportional to the amount of NUP62 and BECN1. The protein concentration of each sample was determined using Bradford reagent (Bio-Rad Protein Assay).

### Detection of Autophagosomes

The neuronal cells were cultured on 96-well plates and after 7 DIV were subjected to ischemia and DIM. The level of autophagosomes was measured by an autophagy assay kit using a proprietary fluorescent autophagosome marker (*λ*_ex_ = 333/*λ*_em_ = 518 nm) as previously described [53]. The autophagosomes were detected using an Infinite M200PRO microplate reader (Tecan, Austria).

### Measurement of HDAC Activity

After experiments the HDAC was detected using a Histone Deacetylase Assay Kit (Sigma–Aldrich, St. Louis, MO, USA) as previously described [54]. The Histone Deacetylase Assay Kit is based on a two-step enzymatic reaction. The first step includes deacetylation of the acetylated lysine side chain by the HDAC-containing sample. The second step is based on the cleavage of the deacetylated substrate by the Developer Solution and the release of the free highly fluorescent group. The fluorescence (*λ*_ex_ = 365 nm/*λ*_em_ = 460 nm) measured by an Infinite M200PRO microplate reader (Tecan, Austria) is directly proportional to the deacetylation activity of the sample. The abovementioned assay kit provides positive and negative controls.

### Immunofluorescence staining of Fas, BCL2, BECN1, NUP62, AhR, CYP1A1, and MAP2

Hippocampal cells were grown on glass cover slips and they were subjected to immunofluorescence double-labeling, as previously described [8]. After a 1 h incubation in a blocking buffer (5% normal donkey serum and 0.3% Triton X-100 in 0.01 M PBS), the cells were treated for 24 h (at 4 °C) with primary antibodies: anti-AhR goat polyclonal (1:50), anti-AhR rabbit polyclonal (1:50), anti-CYP1A1 goat polyclonal (1:50), anti-Fas rabbit polyclonal (1:50), anti-MAP2 rabbit polyclonal (1:100), anti-BCL2 mouse monoclonal (1:50), anti-BECN1 rabbit polyclonal (1:50), anti-NUP62 mouse monoclonal (1:50). The staining with primary antibodies was followed by a 4 h incubation with secondary antibodies, including Cy3-conjugated anti-mouse IgG (1:300), Cy3-conjugated anti-rabbit IgG (1:300), Cy5-conjugated anti-mouse IgG (1:300), Cy3-conjugated anti-goat IgG (1:300). The samples were consequently washed with PBS, mounted, and cover-slipped. They were analyzed using a confocal microscope (DMi8-CS, Leica MicroSystem, Wetzlar, Germany) with a HC Plan-Apochromat CS2 63x/1.4 Oil objective. A two laser lines emitting at 570 and 670 nm, were used to excite the Cy3- and Cy5-conjugated antibodies.

### Data analysis

Statistical data analysis was performed on raw data, which were expressed as the mean arbitrary absorbance or fluorescence units per well containing 62,500 cells (measurements of caspase-3 activity, LDH release, and autophagosome formation), the fluorescence units per 1.5 million cells (HDAC activity and ELISA) or the mean optical density per 30 µg of protein (western blot). One-way analysis of variance (ANOVA) was preceded by Levene’s test to determine the overall significance. Changes between the normoxic control and experimental groups under normoxic conditions were assessed using the post hoc Newman-Keuls test, and statistically significant differences were designated follows: ^*^p < 0.05, ^**^p < 0.01, ^***^p < 0.001 (versus the normoxic control). Alterations between the ischemic control and experimental groups in ischemia were assessed using a post hoc Newman-Keuls test, and significant differences were designated follows: ^#^p < 0.05, ^##^p < 0.01, and ^###^p < 0.001 (versus the ischemic control), ^p < 0.05, p^^^<0.001 (versus the cultures exposed to ischemia and DIM). The results are expressed as the means ± SEM of three to four independent experiments. The number of replicates in each experiment ranged from 7 to 10 except for ELISA and western blot analyses, whose numbers of replicates ranged from 2 to 3. The results of the caspase-3, LDH, and HDAC activity assays were presented as a percentage of the control values to compare the effects of DIM on ischemic neuronal tissues.

## Results

### Effects of ischemia on LDH release and caspase-3 activity in hippocampal cultures at 2, 7 and 12 DIV

**Fig. 1:**
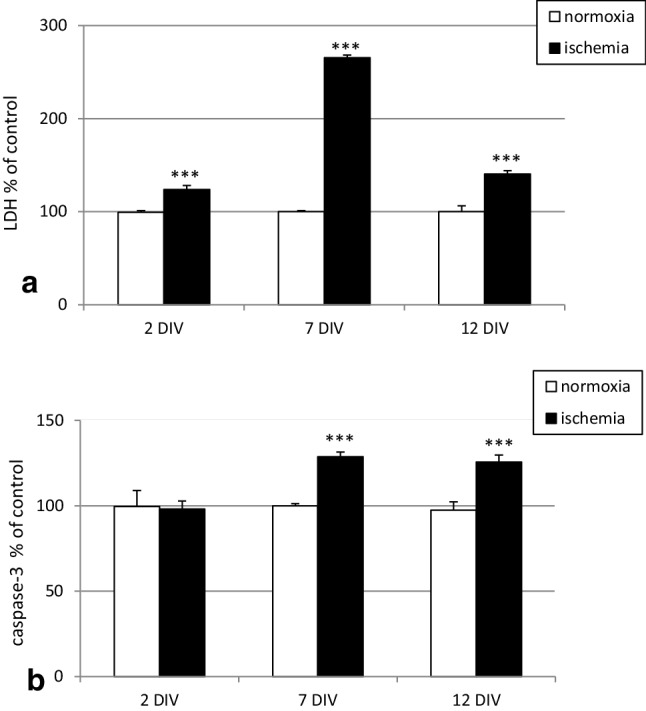
Impact of ischemia on LDH (panel **a**) and caspase-3 (panel **b**) activities in mouse primary hippocampal cell cultures at 2, 7, and 12 DIV. The cells were exposed to ischemia for 6 h and reoxygenation for 18 h. The results are expressed as the percent of the normoxic control values. Each bar in the graph represents the mean ± SEM of three to four experiments. The number of repetitions in each experiment ranged from 7 to 10. ***p < 0.001 versus normoxic cultures

Ischemia increased LDH release from hippocampal cells at 2 DIV by 24% compared to normoxia, which was normalized to 100%, and it did not stimulate caspase-3 activation (Fig. 1, panels a, b). At 7 DIV, the ischemic condition enhanced LDH release to 265% and increased caspase-3 activity to 128% (Fig. 1, panels a, b). At 12 DIV, ischemia increased LDH and caspase-3 activities to 140% and 126%, respectively (Fig. 1panels a, b).

### Inhibitors of caspase-8, caspase-9 and p38 MAPK but not JNK inhibitor reduced the ischemia-induced LDH release and caspase-3 activity in hippocampal cultures at 7 DIV

**Fig. 2:**
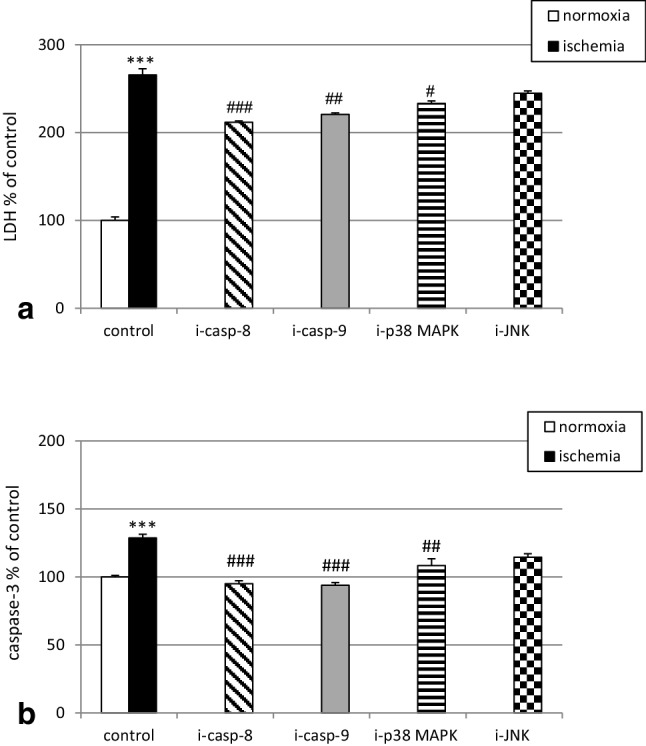
Effects of specific caspase and kinase inhibitors on the ischemia-induced LDH release (panel a) and caspase-3 activity (panel b) in primary cultures of mouse hippocampal cells at 7 DIV. Hippocampal cells were simultaneously treated with inhibitors of caspase-8 (Z-IETD-FMK, 40 µM), caspase-9 (Z-LEHD-FMK, 40 µM), p38 MAP kinase (SB203580, 1 µM) and Jun-N terminal K kinase (SP600125, 1 µM) or vehicle (0.1% DMSO) and ischemia for 6 h. The results are presented as the percent of ischemic control values. Each bar represents the mean ± SEM of three to four independent experiments. The number of replicates for each experiment ranged from 7 to 10. ***p < 0.001 versus normoxic cultures; ^#^p < 0.05, ^##^p < 0.01, ^###^p < 0.001 versus the cultures exposed to ischemia

As mentioned above, ischemia increased LDH release to 265%. In the presence of caspase-8 and caspase-9 inhibitors (40 µM), the ischemia-induced LDH release was diminished to 212% and 220%, respectively. Treatment with a p38 MAPK inhibitor (1 µM) decreased ischemia-stimulated LDH release to 233%. However, exposure to a JNK inhibitor (1 µM) did not significantly affect LDH release in mouse hippocampal cells at 7 DIV (Fig. 2, panel a).

In hippocampal cultures subjected to ischemia, inhibitors of caspase-8, caspase-9, and p38 MAPK decreased caspase-3 activity to 95%, 94% and 108%, respectively. Cotreatment with a JNK inhibitor (1 µM) did not significantly influence the ischemia-induced caspase-3 activity (Fig. 2, panel b).

### DIM inhibited the ischemia-evoked formation of apoptotic nuclei and normalized neuronal cell survival in hippocampal cultures undergoing ischemia at 7 DIV

**Fig. 3:**
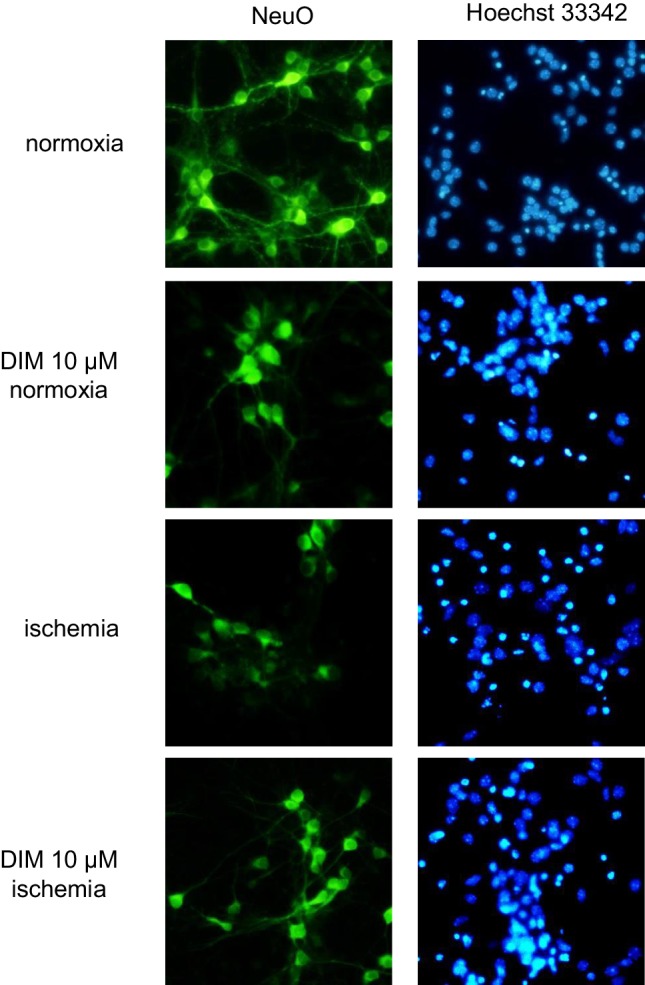
Effects of ischemia and DIM (10 µM) on NeuO (first column) and Hoechst 33342 (second column) staining in mouse hippocampal cultures at 7 DIV. The neurons were cultured on glass cover slips, washed with PBS and exposed to 0.125 µM NeuO at RT for 2 h. The cells were then rewashed and incubated with Hoechst 33342 (0.6 µg/ml) at RT for 5 min. The neurons with light-colored cytoplasm were identified as dying cells, whereas the cells with bright fragmented nuclei showing condensed chromatin were identified as undergoing apoptotic cell death

In the present study, 6 h of ischemia induced apoptosis in mouse hippocampal cells, as indicated by the formation of apoptotic bright fragmented nuclei containing condensed chromatin labeled with Hoechst 33342. Compared with the cells exposed to normoxic conditions, the cells exposed to ischemia exhibited reduced cell survival, as indicated by the decreased fluorescence of NeuO-stained cells. Under ischemic conditions, DIM (10 µM) diminished the number of fragmented nuclei and normalized the ratio of healthy living hippocampal cells. Treatment with DIM (10 µM) under normoxic conditions did not evoke any changes in the cells stained with Hoechst 33342 and NeuO (Fig. 3).

### Effects of DIM on ischemia-induced LDH release and caspase-3 activity in hippocampal cultures at 2 DIV

**Fig. 4:**
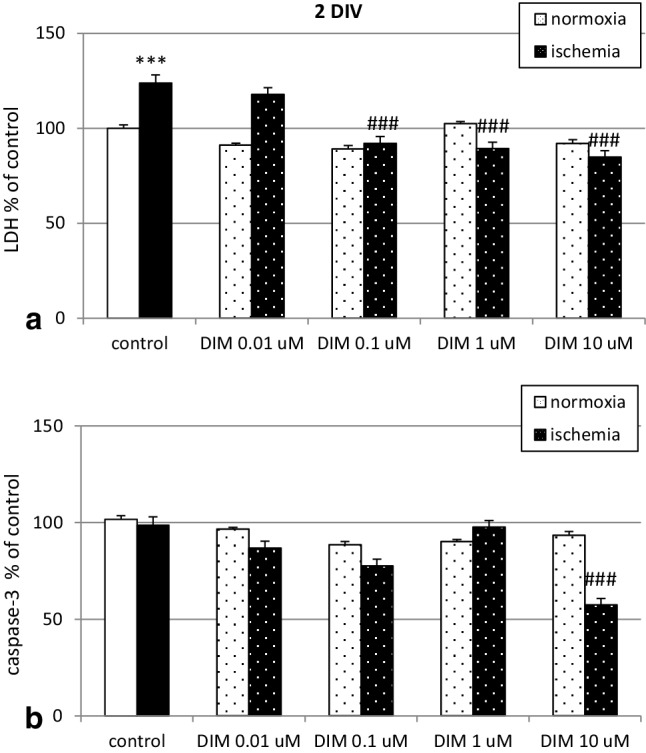
Effects of DIM (0.01-10 µM) on the ischemia-induced (6 h) LDH release (panel **a**) and caspase-3 activity (panel **b**) in primary cultures of mouse hippocampal cells at 2 DIV. Hippocampal cells were simultaneously treated with DIM or vehicle (0.1% DMSO) and ischemia for 6 h. The results are presented as the percentage of ischemic control. Each bar in the graph represents the mean ± SEM of three independent experiments. The number of repeats for each experiment ranged from 7 to 10. ***p < 0.001 versus normoxic cultures; ^###^p < 0.001 versus the cultures exposed to ischemia

6 h of ischemia induced 24% increase in LDH release from mouse hippocampal cells compared to that of normoxia. Treatment with 0.1–10 µM DIM inhibited the effects of ischemia and reduced LDH release to 84–92%. Under normoxic conditions, DIM did not influence the control level of LDH (Fig. 4, panel a). As mentioned above, ischemia did not stimulate caspase-3 activation at 2 DIV. However, DIM at a concentration of 10 µM decreased caspase-3 activity to 57% (Fig. 4, panel b).

### Effects of DIM on ischemia-induced LDH and caspase-3 activities in hippocampal cultures at 7 DIV

**Fig. 5:**
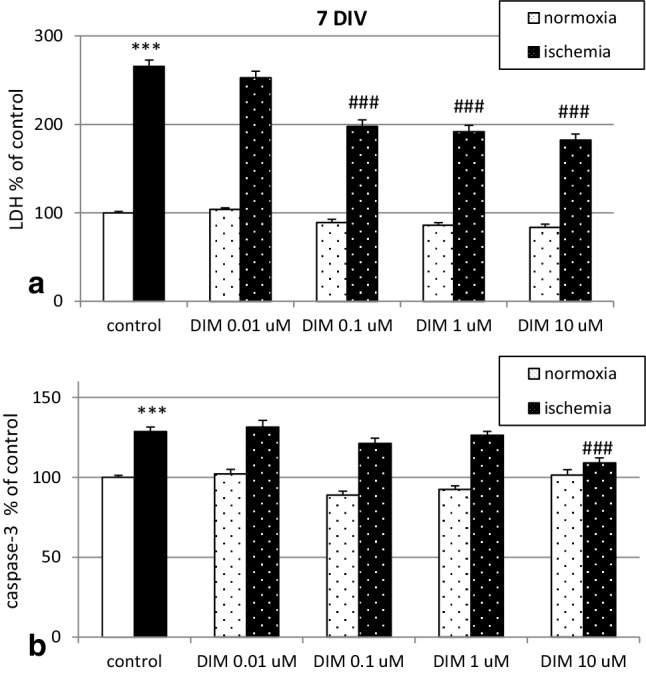
Influence of DIM (0.01-10 µM) on the ischemia-induced (6 h) LDH release (panel **a**) and caspase-3 activity (panel **b**) in primary cultures of mouse hippocampal cells at 7 DIV. Hippocampal cells were simultaneously treated with DIM or vehicle (0.1% DMSO) and ischemia for 6 h. The results are expressed as the percentage of control values. Data represents the mean ± SEM of three to four independent experiments. The total number of replicates for each experiment ranged from 7 to 10. ***p < 0.001 versus normoxic cultures; ^###^p < 0.001 versus the cultures exposed to ischemia

In our model, ischemia increased LDH release to 265% of the normoxic control level. At concentrations ranging from 0.1 to 10 µM, DIM inhibited this effect and reduced LDH release to levels ranging from 182 to 197%. Under normoxic conditions, DIM did not change the control level of LDH (Fig. 5, panel a). At 7 DIV, ischemia induced caspase-3 activity to 129% of the normoxic control value. Treatment with 10 µM DIM decreased ischemia-stimulated activation of caspase-3 to 108% of the ischemic control value (Fig. 5, panel b).

### Effects of DIM on ischemia-induced LDH release and caspase-3 activity in hippocampal cultures at 12 DIV

**Fig. 6:**
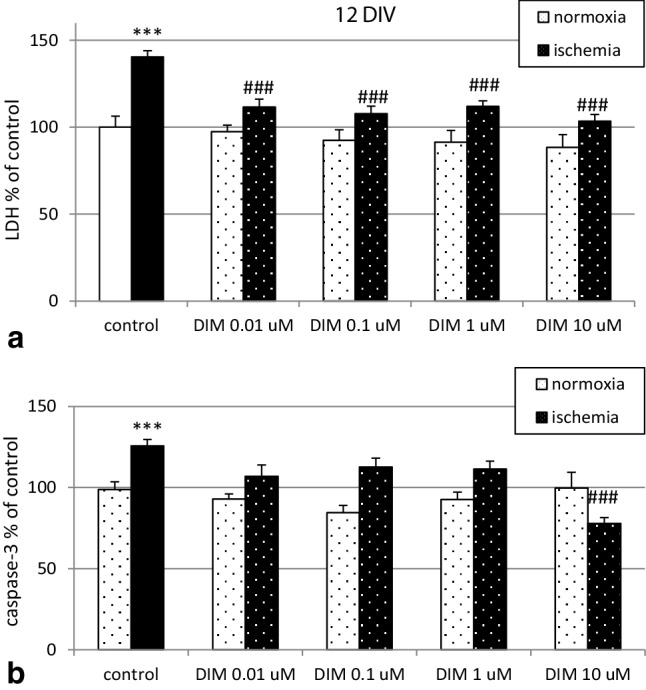
Influence of DIM (0.01–10 µM) on the ischemia-induced (6 h) LDH release (panel **a**) and caspase-3 activity (panel **b**) in primary cultures of mouse hippocampal cells at 12 DIV. Hippocampal cells were simultaneously treated with DIM or vehicle (0.1% DMSO) and ischemia for 6 h. The results are presented as a percent of the control. The bars represent the mean ± SEM of three experiments. In each experiment the number of replicates ranged from 7 to 10. ***p < 0.001 versus normoxic cultures; ^###^p < 0.001 versus the cultures exposed to ischemia

Six hours of ischemia increased LDH release to 140% of the normoxic control level. Treatment with 0.01-10 µM DIM reduced ischemia-evoked LDH release to 103–107% (Fig. 6, panel a). At 12 DIV, ischemia increased caspase-3 activity to 126% compared to that in the normoxic control. Treatment with 10 µM DIM decreased the ischemia-evoked activation of caspase-3 to 78% (Fig. 6, panel b).

### Inhibitors of caspase-8 and p38 MAPK but not caspase-9 inhibitor reduced the ischemia-induced LDH release and caspase-3 activity in hippocampal cultures at 7 DIV

**Fig. 7:**
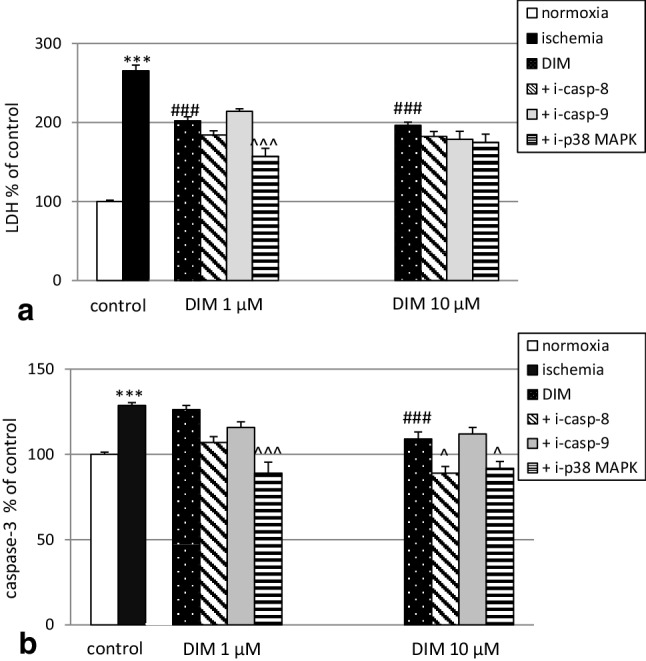
Effects of DIM (1, 10 µM) and inhibitors of caspase-8, caspase-9 and p38 MAPK kinase on ischemia-induced (6 h) LDH release (panel a) and caspase-3 activity (panel b) in primary cultures of mouse hippocampal cells at 7 DIV. Hippocampal cells were simultaneously treated with DIM and inhibitors followed by ischemia for 6 h. The results are expressed as the percentage of control values. Data on the bars represents the mean ± SEM of three to four independent experiments. The number of repeats for each experiment ranged from 7 to 10. ***p < 0.001 versus normoxic cultures; ^###^p < 0.001 versus the cultures exposed to ischemia; ^^^p < 0.05 and ^^^^^p < 0.001 versus the cultures exposed to ischemia and DIM

In our study, DIM decreased the ischemia-induced LDH release to 202% of the ischemic control level. Addition of p38 MAPK inhibitor enhanced the neuroprotective potential of DIM and decreased LDH release to 157%. The presence of caspase-8 and caspase-9 inhibitors (40 µM) did not influence the effect of DIM with respect to LDH release (Fig. 7, panel a).

Under ischemic conditions, DIM (10 µM) reduced ischemia-stimulated caspase-3 activity to 108%. Treatment with caspase-8 (40 µM) and p38/MAPK (1 µM) inhibitors decreased caspase-3 activity to 89% and 91%, respectively. However, exposure to a caspase-9 inhibitor (40 µM) did not significantly affect caspase-3 activity in mouse hippocampal cells at 7 DIV (Fig. 7, panel b).

### DIM inhibited the ischemia-stimulated levels of Caspase-3 and Fas but not BAX protein in hippocampal cells

**Fig. 8:**
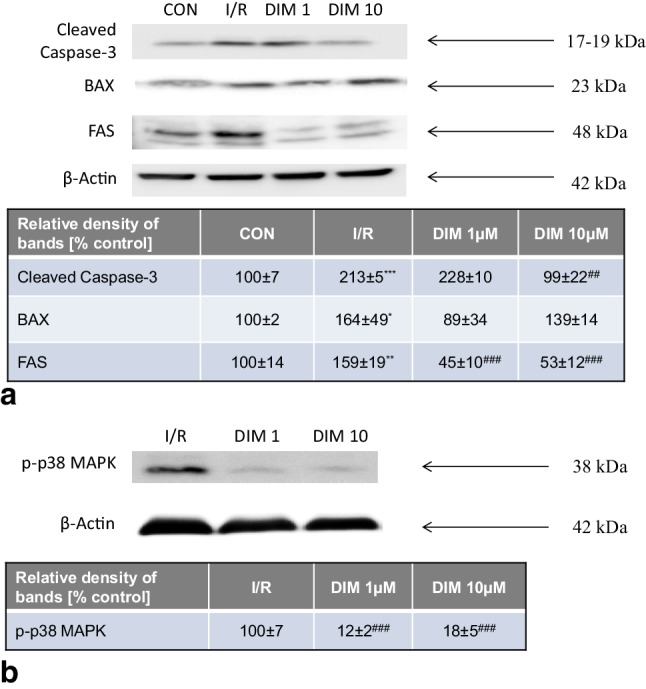
Influence of ischemia and DIM (1, 10 µM) on the changes in relative levels of Caspase-3, BAX and Fas proteins (panel **a**) as well as the level of phosphorylated p38 MAP kinase (panel **b**) in hippocampal cultures at 7 DIV. Hippocampal cultures were simultaneously treated with DIM (1, 10 µM) and ischemia for 6 h. Protein samples were collected from hippocampal cultures after 18 h of reoxygenation, denatured, subjected to electrophoresis, transferred to PVDF membrane and immunolabeled. The images were visualized using a Luminescent Image Analyzer Fuji-Las 4000 (Fuji, Japan). Immunoreactive bands were quantified using an Multi-Gauge-V3.0 software (Fujifilm, France) and the relative protein levels of cleaved Caspase-3, Fas, BAX and p38 MAPK were presented as a percentage of the control levels. Each value represents the mean of three independent experiments ± SEM. The number of replicates in each experiment ranged from 2 to 3. *p < 0.05, **p < 0.01, and ***p < 0.001 versus normoxic cultures; ^##^p < 0.01, ^###^p < 0.001 versus ischemia-treated cultures

Ischemia followed by reoxygenation increased the relative protein levels of Caspase-3, BAX, and Fas by 59–113%. DIM (1–10 µM) inhibited the ischemia-stimulated levels of Caspase-3 and Fas but did not change the protein level of BAX (Fig. 8, panel a). Moreover, DIM greatly decreased the protein level of p-p38 MAPK to 12–18% of the ischemic control level (Fig. 8, panel b). All samples had the same protein concentrations, as confirmed by the expression of β-Actin (loading control) (Fig. 8).

### Effects of ischemia and ischemia followed by reoxygenation on autophagy-related protein concentration levels

**Fig. 9:**
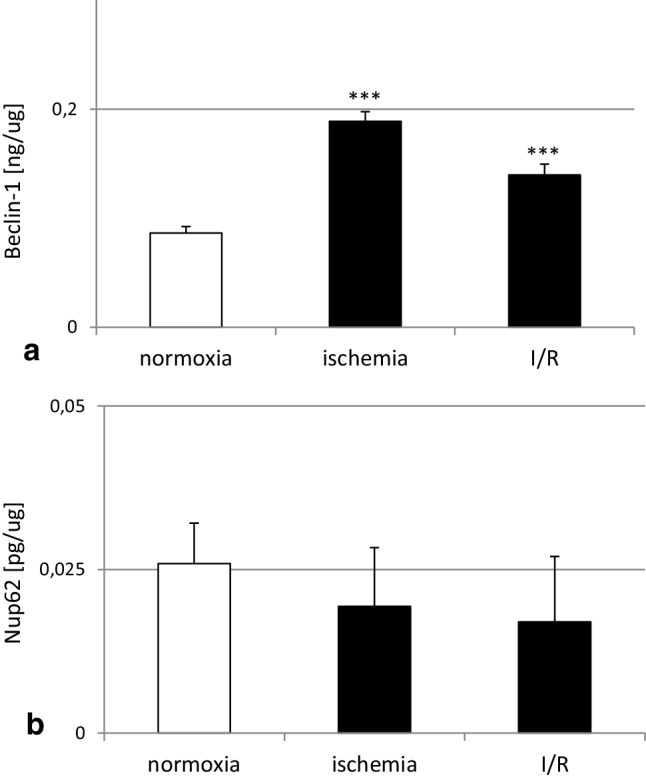
Effects of ischemia and ischemia followed by reoxygenation on changes in the concentration levels of BECN-1 (panel a) and NUP62 (panel b) protein in hippocampal cultures at 7 DIV. Hippocampal cultures were subjected to 6 h of ischemia or ischemia followed by 18 h reoxygenation. The BECN-1 and NUP62 concentrations were measured via specific ELISA and presented as the ng per µg of BECN-1 or pg per µg of NUP62 of total nondenatured protein. Each bar or value represents the mean of three independent experiments ± SEM. The number of replicates in each experiment ranged from 2 to 3. ***p < 0.001 versus normoxic cultures

The protein concentration level of Beclin-1 (BECN1) in hippocampal cells under normoxic conditions reached 0.09 ng/µg. Six hours of ischemia increased the level of Beclin-1 to 0.18 ng/µg with respect to the normoxic condition. Ischemia followed by reoxygenation stimulated the concentration level of Beclin-1 to 0.14 ng/µg (Fig. 9, panel a).

Under normoxic conditions, the level of NUP62 reached 0.026 pg/µg. Ischemia and ischemia followed by reoxygenation did not change the level of NUP62 (Fig. 9, panel b).

### DIM normalized the ischemia-reduced level of NUP62 and decreased the protein levels of LC3 and BECN1 in hippocampal cells

**Fig. 10:**
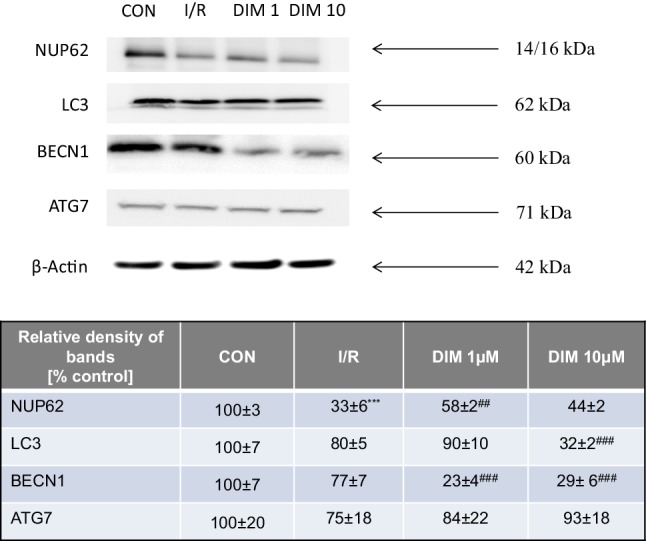
Influence of ischemia and DIM (1, 10 µM) on the changes in relative levels of autophagy-related proteins in hippocampal cultures at 7 DIV. Hippocampal cultures were simultaneously treated with DIM (1, 10 µM) and ischemia for 6 h. Protein samples were collected from hippocampal cultures after 18 h of reoxgenation, denatured, subjected to electrophoresis, transferred to PVDF membrane, and immunolabeled. The membranes were developed using a Luminescent Image Analyzer Fuji-Las 4000 (Fuji, Japan). Immunoreactive bands were quantified using an MultiGauge V3.0 software (Fujifilm, France), and the relative protein levels of NUP62, LC3, BECN1 and ATG7 were presented as a percentage of the control levels. Each value represents the mean of three independent experiments ± SEM. The number of replicates in each experiment ranged from 2 to 3. ***p < 0.001 versus normoxic cultures; ^##^p < 0.01, ^###^p < 0.001 versus ischemia-treated cultures

Ischemia followed by reoxygenation decreased the NUP62 protein level to 33% but did not influence the levels of LC3, BECN1 and ATG7. DIM (1–10 µM) normalized the level of NUP62 reduced by ischemia, and it decreased the protein levels of LC3 and BECN1 to 23–32% (Fig. 10). All samples had identical protein concentrations, as verified by the expression of β-Actin (loading control) (Fig. 10).

### Effects of ischemia and DIM on autophagosome detection in hippocampal and neocortical cells

**Fig. 11:**
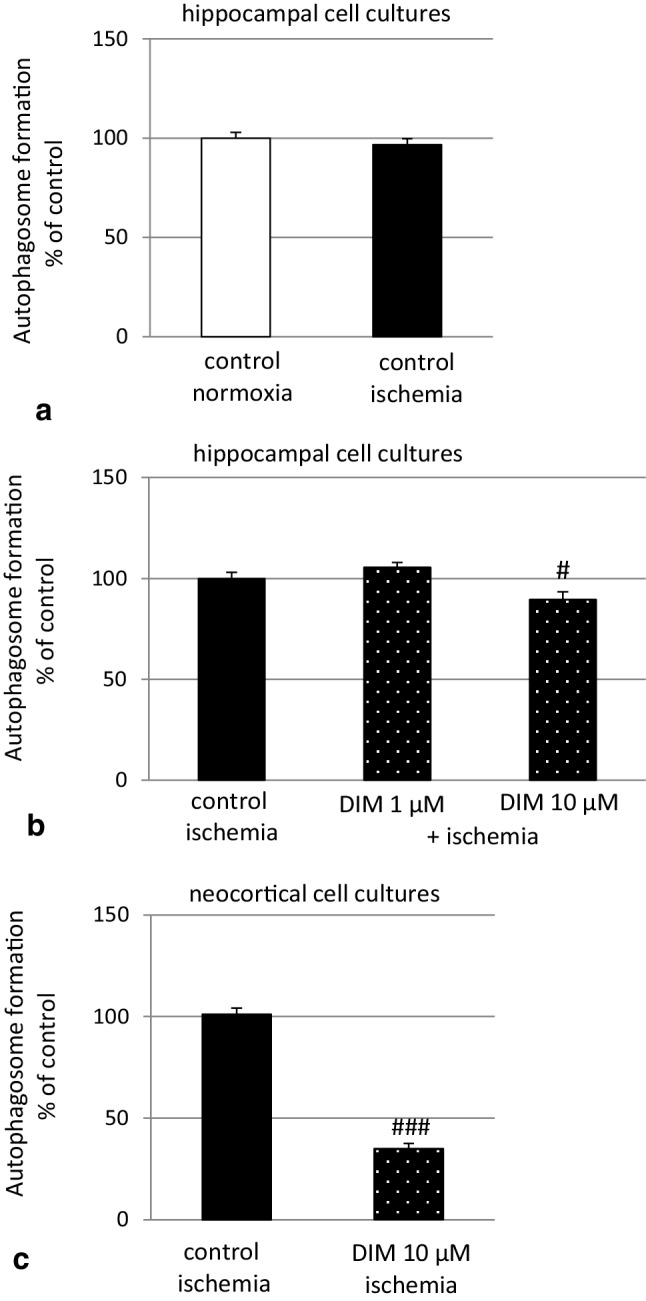
Effect of ischemia with reoxygenation and DIM (1, 10 µM) on autophagosome formation in hippocampal (panel **a** and **b**) and neocortical cultures (panel **c**) at 7 DIV. Neuronal cultures were simultaneously treated with DIM (1, 10 µM) and ischemia (6 h) followed by reoxygenation (18 h). The data are expressed as the mean ± SEM of three independent experiments consisting of eight replicates per treatment group. ^#^p < 0.05 and ^###^p < 0.001 versus the ischemic control

Exposure of hippocampal cultures to 6 h of ischemia followed by 18 h of reoxygenation did not change the level of autophagosomes (Fig. 11, panel a). In hippocampal cells subjected to ischemia, DIM (10 µM) decreased the autophagosome level by only 11% (Fig. 11, panel b). However, in neocortical cells undergoing ischemia, DIM reduced autophagosome formation to 34% of the ischemic control value (Fig. 11, panel c).

### DIM normalized the ischemia-reduced HDAC activity in hippocampal cell cultures

**Fig. 12:**
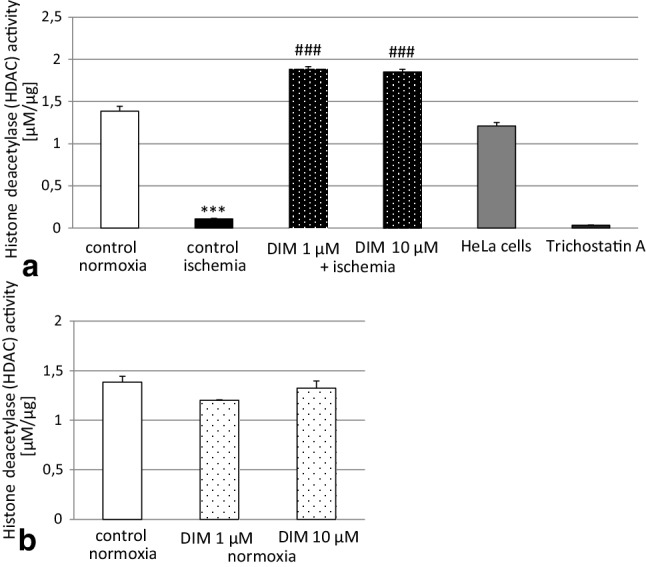
Effects of ischemia and DIM on HDAC activity in primary cultures of mouse hippocampal cells at 7 DIV. The cells were subjected to ischemia alone or in combination with DIM for 6 h. The results are presented as a percentage of the normoxic control values. The bars represent the mean of three independent experiments ± SEM. The number of replicates in each experiment ranged from 5 to 8. ***p < 0.001 versus normoxic control cultures, ^###^p < 0.001 versus ischemic control cultures

In hippocampal cells under normoxia, the level of HDAC activity reached 1.38 µM/µg. A 6 h exposure of hippocampal cultures to ischemia followed by 18 h of reoxygenation reduced the level of HDAC activity to 0.1 µM/µg. Treatment with DIM (1–10 µM) increased the activity of HDAC to 1.88 and 1.84 µM/µg (Fig. 12, panel a). Under normoxic conditions, DIM did not change the HDAC activity (Fig. 12, panel b).

### DIM impaired AhR but not ERα signaling pathways in hippocampal neurons undergoing ischemia

**Fig. 13:**
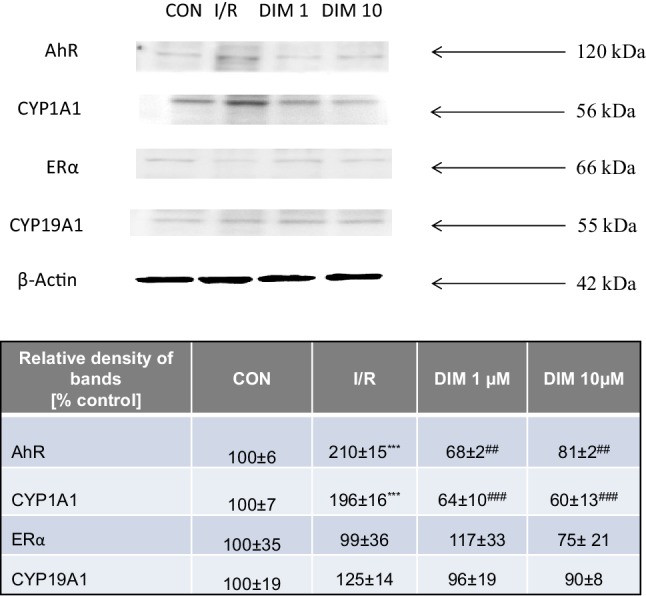
Effects of ischemia and DIM on changes in the protein expression levels of AhR, CYP1A1, ERα, CYP19A1 in hippocampal cultures at 7 DIV. Hippocampal cultures were simultaneously treated with DIM (1, 10 µM) and ischemia for 6 h. Protein samples were collected from hippocampal cultures after 18 h of reoxygenation, denatured, electrophoretically separated, transferred to PVDF membrane, and immunolabeled. The signals were developed using a Luminescent Image Analyzer Fuji-Las 4000 (Fuji, Japan). Immunoreactive bands were quantified using an image analyzer (ScienceLab, MultiGauge V3.0), and the relative protein levels of AhR, CYP1A1, ERα and CYP19A1 were presented as a percentage of the control. ***p < 0.001 versus normoxic cultures; ^##^p < 0.01, ^###^p < 0.001 versus ischemia-treated cultures

Ischemia followed by reoxygenation increased the AhR and CYP1A1 protein levels to about 200%, but did not influence the levels of ERα and CYP19A1. DIM (1–10 µM) decreased the protein levels of AhR and CYP1A1 to 60–81%. However, it did not affect the protein levels of ERα and CYP19A1. All samples had equal protein concentrations, as proved by the expression of β-actin (loading control) (Fig. 13).

### Effects of ischemia and DIM on the AhR, CYP1A1, Fas, BCL2, BECN1, NUP62 and MAP2 cellular distribution in hippocampal cells

**Fig. 14:**
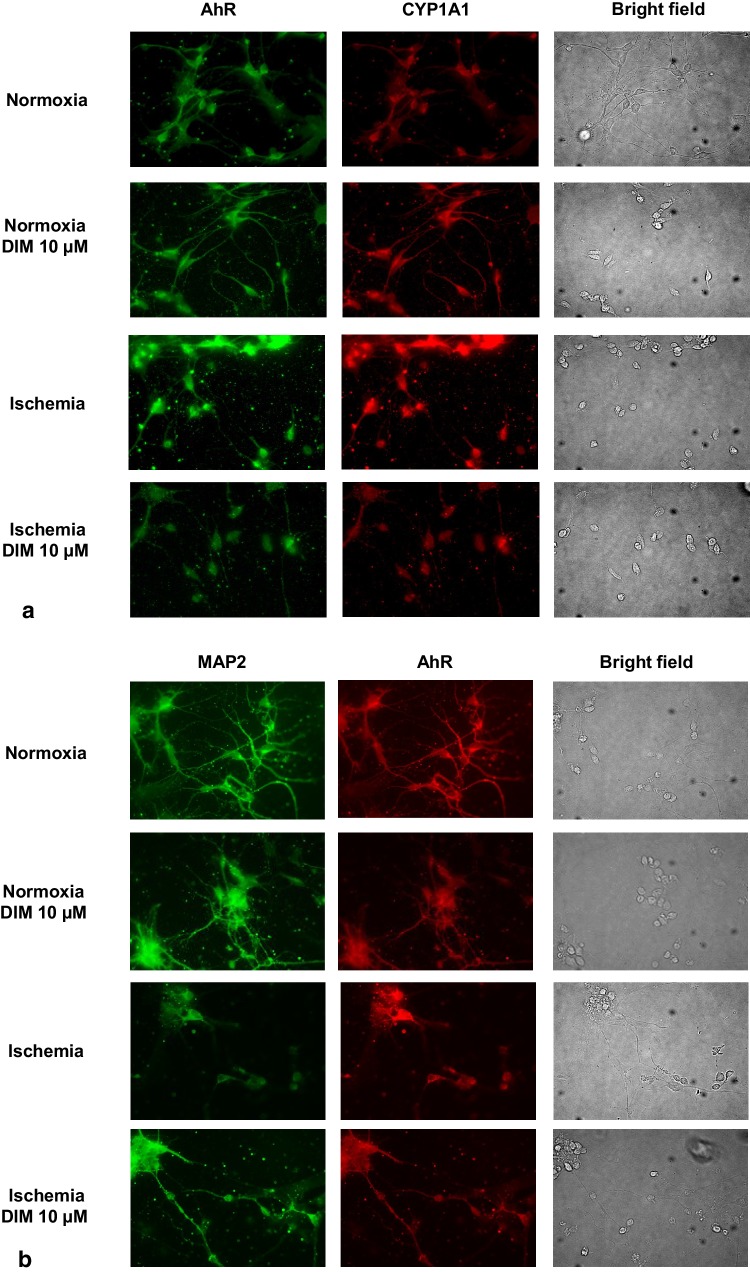

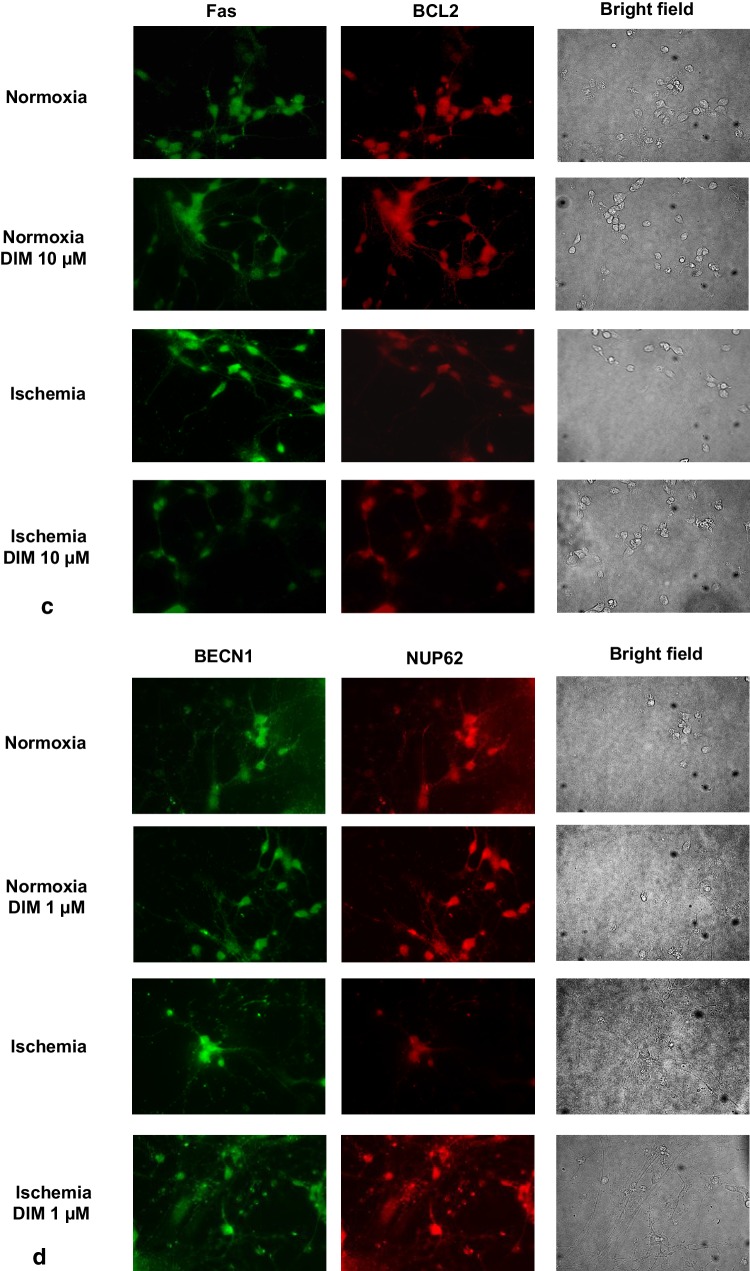
Impact of ischemia (6 h) and DIM (1,10 µM) on the cellular distribution of AhR (panel **a**—green, panel **b**—red), CYP1A1 (panel **a**—red), BECN1 (panel **d**—green) NUP62 (panel **d**—red), Fas (panel **c**—green), BCL2 (panel **c**—red) and MAP2 (panel **b**—green) in hippocampal cultures. Primary hippocampal cultures were treated with either ischemia alone or in combination with DIM for 6 h. The cells were cultured on glass cover slips and subjected to immunofluorescence double-labeling. The samples were analyzed using a confocal microscope (DMi8-CS, Leica MicroSystem, Wetzlar, Germany) with a HC Plan-Apochromat CS2 63x/1.4 Oil objective

Immunofluorescence labeling showed the expression of AhR, CYP1A1, Fas, BCL2, BECN1, NUP62 and MAP2 in mouse hippocampal cells in primary culture. In Fig. 14, exposure to ischemic conditions increased specific immunofluorescence for AhR (panel a—green, panel b—red), AhR-regulated CYP1A1 (panel a—red), Fas (panel c—green), and BECN1 (panel d—green). Ischemia decreased specific immunolabeling for BCL2 (panel c—red), NUP62 (panel d—red), and MAP2 (panel b—green). DIM (1, 10 µM) partially normalized ischemia-induced changes i.e., it reduced the AhR, CYP1A1, Fas, and BECN1 immunolabeling. DIM also increased NUP62 and MAP2, but not BCL2 immunostaining. Under normoxic conditions, DIM (1, 10 µM) did not affect the cellular levels of the selected proteins. Based on the results from western blot, 10 µM DIM was used for the immunolabeling for the AhR, CYP1A1, Fas, BCL2, and MAP2, whereas 1 µM DIM was used for the immunolabeling for BECN1 and NUP62.

## Discussion

For the first time, the results of the present study demonstrated that 3,3′-diindolylmethane (DIM) protects neurons against ischemia-induced damage at earlier and later stages of neuronal development, as evidenced at 2, 7 and 12 DIV. In comparison to our previously published model of hypoxia, our newly developed model of OGD reflects more extensively the features of stroke. These include ischemia-induced apoptosis and autophagy and possibly corresponds to ischemia-evoked disruption of HDAC activity and AhR/CYP1A1 signaling pathway. In the present study, DIM partially reversed OGD-induced apoptosis, autophagy and AhR/CYP1A1 signaling as well as OGD-inhibited HDAC activity. However, DIM did not affect ERα/CYP19A1 signaling in mouse neurons subjected to OGD, which points to ERα-independent mechanisms of neuroprotective action of DIM against ischemia.

Since the most profound effect of ischemia was observed at 7 DIV, this *in vitro* stage of development was chosen for the subsequent experiments. In a newly developed model of OGD (6 h of ischemia followed by 18 h of reoxygenation), we observed activation of caspase-3 that was followed by an increase in BAX, Fas and Caspase-3 protein levels, LDH release and neuronal cell death. In our study, OGD-evoked apoptotic cell death was additionally supported by apoptotic fragmentation of nuclei as demonstrated by Hoechst 33342 staining and by an increase in Fas and decrease in BCL2 as evidenced by western blot and immunofluorescence labeling. Neuronal nature of hippocampal cells was confirmed by the MAP2 staining. In our model, OGD-induced apoptosis was accompanied with an increase in protein expression levels of AhR and AhR-related CYP1A1 but not in the levels of ERα and ERα-related CYP19A1. In addition, the cellular distribution of AhR and CYP1A1 was evidenced by the fluorescence microscopy. Similarly to our research, Liu et al. [55] showed an increase in chromatin condensation as well as in caspase-3 cleavage in rat hippocampal cell cultures subjected to OGD. Moreover, Cuartero et al. [7] demonstrated increased immunofluorescence of AhR in rat cortical neurons after ischemia in vitro. Our newly developed model of OGD reflects the ischemia-induced effects, including LDH release and cell death that were observed by other researchers in cortical and hippocampal cells as well as in brain slices subjected to OGD [56–58]. Our data are also in line with other authors who have demonstrated ischemia-induced increases in Fas, Caspase-3, BAX and AhR protein levels in in vitro and in vivo models of stroke [7, 59–61]. Similarly to present data, our previous results did not show any change in protein expression level of ERα in hippocampal cells undergoing hypoxia *in vitro* [11].

In this study, we demonstrated that DIM partially inhibited ischemia-induced caspase-3 activity and nuclear fragmentation as well as diminished protein expression levels of Fas and Caspase-3. DIM did not influence the ischemia-stimulated level of BAX as well as it did not affect the ischemia-decreased BCL2 immunostaining. One may suggest that the neuroprotective action of DIM against OGD is associated with inhibition of the extrinsic apoptotic pathway. Indeed, specific inhibitors of caspase-8, but not caspase-9, enhanced the neuroprotective capacity of DIM, which supports our hypothesis. In our previous study, we showed that DIM decreased hypoxia-induced caspase-3 activity and nuclear condensation in hippocampal cells undergoing hypoxia [8]. Recently, it has been demonstrated that DIM and its analog (DIM-CpPhtBu) inhibited apoptosis including apoptotic body formation and caspase activity in cellular models of Parkinson’s disease [13, 17]. In the present model of ischemia, a specific inhibitor of p38 MAPK (SB203580) decreased the ischemia-induced LDH and caspase-3 activities, and these effects were potentiated by DIM. In addition, DIM strongly reduced the protein level of p38 MAPK, which points to involvement of inhibition of this kinase in the neuroprotective action of DIM during ischemia. However, there are no other relevant studies supporting the involvement of p38 MAPK signaling in the neuroprotective effects of DIM. In our study, after treatment of the hippocampal cells with a JNK inhibitor (SP600125), we did not observe inhibition of OGD-induced cell damage. Although Fan et al. [62] noticed that SP600125 reduced damage in rat neuronal cells undergoing ischemia for 30 min, these authors applied the JNK inhibitor as a 30 min pretreatment rather than as a cotreatment as we did in our study. Since AhR promotes apoptosis, controls ERα, and is highly expressed in brain tissue in experimental models of stroke [7, 49, 63], we hypothesize that anti-apoptotic action of DIM against OGD is mediated via inhibition of AhR/CYP1A1 and/or stimulation of ERα/CYP19A1 signaling pathways. In the present study, we demonstrated that DIM inhibited OGD-induced AhR/CYP1A1 protein expression levels including their cellular distribution, but it did not influence the levels of ERα/CYP19A1. There are no relevant studies on the role of ERα signaling pathway in neurons subjected to ischemia and DIM. The only data comes from breast cancer cell line MCF7 where DIM activated or inhibited the estrogen receptor ERα signaling pathway in concentration-dependent manner [64, 65]. Our previous data have shown that DIM protected mouse hippocampal cells against hypoxia via inhibition of AhR signaling [8] which is in line with DIM-evoked neuroprotection against OGD observed in the present study.

In addition to the OGD-induced features of apoptotic and necrotic cell death, our model includes autophagy-related processes. Autophagy is known to involve ATG7, which is essential for initiating autophagy. Beclin-1 promotes lipidation of LC3-I to generate LC3-II, which is localized at the autophagosome membrane. The formation of autophagosomes is inversely correlated to a cargo adaptor NUP62, which binds directly to LC3 and is degraded by autophagy [47, 66]. Although autophagy exerts mainly neuroprotective effects, it is detrimental when massive excitotoxicity and stroke are present. In our study, ischemia evoked a significant decrease in protein levels of NUP62 and an increase in BECN1 as evidenced by western blot, ELISA and immunofluorescence labeling. However, in our model OGD did not affect formation of autophagosomes and protein levels of ATG7 and LC3. Similar to our results, Jiang et al. [67] observed a reduction in the protein level of NUP62 and an increase in the protein level of BECN1 in cellular (PC12 cells) and animal (middle cerebral artery occlusion) models of ischemia. In line with our study, Perez-Rodriguez et al. [68] did not notice a change in the protein level of ATG7 in hippocampal slices subjected to OGD. There are controversies about the role of autophagy in ischemia and expression of relevant markers in various stroke models. Shi et al. [69] demonstrated that morphological features of autophagosomes in cortical cells subjected to OGD appeared only after 72 h of reoxygenation. Moreover, Fan et al. [62] observed an increase in LC3 after 0.5–2 h, but not after 6 h, of reoxygenation in rat cortical cells subjected to OGD. Based on these studies, one may assume that there is no perfect model of ischemia that fully reflects all the features of autophagy, including ATG7, LC3 and autophagosome formation.

Apart from the demonstration that the neuroprotective capacity of DIM involves an inhibition of OGD-induced apoptosis, here, we demonstrated for the first time that DIM also has the capacity to inhibit autophagy. In hippocampal cells undergoing ischemia, DIM partially reversed the ischemia-induced decrease in NUP62, reduced the protein levels of BECN1, LC3 and inhibited autophagosome formation as detected by western blot and/or immunofluorescence staining. There is no other research showing the impact of DIM on OGD-associated autophagy in neuronal cells. The only available data came from MDA-MB-231 and T47D cancer cell lines where DIM (1–10 µM) inhibited H_2_O_2_-induced autophagy, i.e., it inhibited the expression of Beclin-1 and reduced the percentage of cells exhibiting autophagy and conversion of LC3-I into LC3-II [70].

A growing body of research evidence points to a key role of epigenetic modifications in the regulation of the cell death/cell survival ratio during stroke [71]. In our study, we observed a substantial decrease in HDAC activity in hippocampal cells undergoing OGD. HDACs are involved in epigenetic modifications related to deacetylation of histones. Although there is evidence of HDAC stimulation in response to ischemia and although HDAC inhibitors have been postulated as an experimental neuroprotective strategy, there are also data showing inhibition of HDAC in various models of ischemia [28–31]. Our results are in line with those of Baltan et al. [30] and Chen et al. [31], who demonstrated a drastically reduced level of HDACs in mouse brains subjected to transient MCAO. Moreover, Kim et al. [72] showed that inhibition of HDAC1 activity induced DNA double-strand breaks and neuronal cell death, whereas overexpression of HDACs decreased the percentage of neurons undergoing apoptosis and inhibited neurodegeneration in the striatum of rats subjected to ischemia. Here, we have shown that treatment with DIM completely reversed the OGD-induced decrease in HDAC activity, which is epigenetic modification-related enzyme. Based on these results, one may suggest that DIM, which strongly increased HDAC activity in our study, prevents ischemia-induced apoptosis and cell death via epigenetic modification and possibly reduced histone acetylation.

In conclusion, we demonstrated for the first time the strong neuroprotective capacity of DIM in hippocampal cells exposed to ischemia at early and later stages of neuronal development. The protective effects of DIM were mediated via inhibition of ischemia-induced apoptosis and autophagy that was accompanied by a decrease in AhR/CYP1A1 signaling and an increase in HDAC activity. DIM inhibited protein expression of pro-apoptotic factors, i.e., Fas, Caspase-3, and p38 MAPK. DIM also reduced protein levels of autophagy-related BECN1 and LC3, partially reversed the ischemia-induced decrease in NUP62 and inhibited autophagosome formation. The changes in the protein expression levels were detected by western blot, ELISA and immunofluorescence labeling. In addition, DIM increased HDAC activity in hippocampal cells subjected to ischemia. For the first time, this study demonstrated that the neuroprotective action of 3,3′-diindolylmethane against ischemia involves an inhibition of apoptosis and autophagy and depends on AhR/CYP1A1 signaling and HDAC activity, thus providing prospects for the development of new therapeutic strategies that target neuronal degeneration at specific molecular levels.

## Abbreviations

Ac-DEVD-*p*NA: N-acetyl-asp-glu-val-asp *p*-nitro-anilide
AhR: Aryl hydrocarbon receptor
ARNT: Aryl hydrocarbon receptor nuclear translocator
BECN1: Beclin-1
CO_2_: Carbon dioxide
DIM: 3,3′-diindolylmethane
DIV: Days *in vitro*
DMSO: Dimethyl sulfoxide
ELISA: Enzyme-linked immunosorbent assay
ERα: Estrogen receptor alpha
Fas: Fas Cell Surface Death Receptor
FBS: Fetal bovine serum
GFAP: Glial fibrillary acidic protein
HDAC: Histone deacetylase
I: Ischemia
I/R: Ischemia following reoxygenation
JNK: c-Jun N-terminal kinase
LC3: Microtubule-associated proteins 1A/1B light chain
LDH: Lactate dehydrogenase
MAP2: Microtubule-associated protein 2
N_2_: Nitrogen
NeuO: NeuroFluor fluorescent probe
NUP62: Nucleoporin 62
OGD: Oxygen and glucose deprivation
p38 MAPK: p38 mitogen-activated protein kinase
PBS: Phosphate-buffered saline
p-p38 MAPK: Phosphorylated p38 mitogen-activated protein kinase
R: Reoxygenation
RT: Room temperature
